# Obstetric delivery in mechanically ventilated critically ill pregnant women

**DOI:** 10.1186/2197-425X-3-S1-A275

**Published:** 2015-10-01

**Authors:** SE Lapinsky, JA Rojas-Suarez, TM Crozier, DN Vasquez, N Barrett, G Bourjeily

**Affiliations:** Mount Sinai Hospital, Toronto, Canada; University of Toronto, Interdepartmental Division of Critical Care Medicine, Toronto, Canada; Gestion Salud Clinic, Intensive Care Unit, Cartagena, Colombia; Universidad de Cartagena, Grupo de Investigación en Cuidados Intensivos y Obstetricia, Cartagena, Colombia; Monash Medical Centre, Intensive Care Unit, Clayton, Australia; Sanatorio Anchorena, Buenos Aires, Argentina; Monash Medical Centre, Department of Obstetrics & Gynaecology, Clayton, Australia; Pulmonary and Critical Care Medicine, Warren Alpert Medical School of Brown University, Providence, RI United States; The Miriam Hospital, Providence, RI United States

## Introduction

Approximately 1 in 500 pregnancies develop complications requiring mechanical ventilatory support, the majority of episodes occurring postpartum. While uncommon, the situation of a pregnant woman requiring ventilatory support for respiratory failure is very concerning. Delivery of the fetus is often considered, either to improve maternal well-being or due to concerns for the fetus. Little data exist to guide physicians in these decisions.

## Objectives

To evaluate the effect of obstetric delivery on maternal respiratory function, in pregnant women ventilated for respiratory failure.

## Methods

Subgroup analysis of a retrospective review of pregnant women from 4 ICUs in 4 countries over a 10 year period, who received mechanical ventilation for greater than 24 hours. In women who delivered while on mechanical ventilation, maternal respiratory parameters were evaluated pre-delivery and 2-5 hours and 12-15 hours post-delivery. Respiratory system compliance was estimated from recorded ventilator plateau pressure and tidal volume. Data are presented as median and interquartile ratio (IQR).

## Results

We identified nine women who delivered while ventilated for respiratory failure. Median age was 29 (22.5-33.5) years and gestation 29 (27.5-31) weeks. Pre-delivery Pa02/Fi02 ratio was 268 (131-317) and PaC02 37.3 (33.2-49) mmHg. Duration of ventilation prior to delivery was 1.5 (1-2) days, and time from delivery to extubation was 2 (1-3.8) days. All deliveries were by Cesarean section except one spontaneous stillborn delivery at 26 weeks. Cesarean sections were performed for either maternal respiratory indications (n = 3) or for obstetric/fetal indications (n = 5). APGAR scores at 1 minute were low (≤7) in all 8 elective deliveries, and only 3 recovered to >7 by 5 minutes. All mothers survived, as did all but one neonate (the spontaneous delivery).

Delivery had a variable effect on maternal respiratory parameters. By 12-15 hours, oxygenation index had improved from a median 7.5 (5.9-16.2) to 6 (4.9-9.1), and PEEP was reduced from 11 cmH_2_O (8.5-12) to 9 cmH_2_O (6.5-10). Estimated compliance improved in most patients, but to a variable degree. Three hypercapnic patients (mean C02 57 mmHg) improved, to mean PaC02 of 46 mmHg.

## Conclusions

This relatively small case series examines a rare but important intervention in the ICU - delivery of the fetus in a pregnant woman ventilated for respiratory failure. Delivery occurred early in the mothers' ventilator course, followed by extubation within a few days in the majority of patients. The effect of delivery on maternal respiratory function was variable, with some, but not all women showing an improvement in oxygenation and respiratory system compliance. At delivery, fetal compromise was evident but all neonates survived to hospital discharge.

